# Introducing a novel mean-reverting Ornstein–Uhlenbeck process based stochastic epidemic model

**DOI:** 10.1038/s41598-024-52335-6

**Published:** 2024-01-22

**Authors:** Parisa Nabati

**Affiliations:** Faculty of Science, Urmia University of Technology, Urmia, Iran

**Keywords:** Applied mathematics, Diseases

## Abstract

The major objective of this paper is to examine a novel mean-reverting Ornstein–Uhlenbeck process-based stochastic SIRD model for transmission the epidemic disease that is a great crisis in numerous societies. For this purpose, the deterministic model is further converted into the stochastic form by allowing the infection rate satisfies the mean-reverting Ornstein–Uhlenbeck process to account the uncertainties involved in epidemic spread. At first using Lyapunov functions, the solution’s uniqueness and positivity will be demonstrated. Subsequently, the stochastic epidemic threshold $\Re_{0}^{S}$ that controls the disease’s extinction and persistence in the mean is identified analytically. It has been established that when $\Re_{0}^{S} < 1$ the disease will extinguish, whereas if $\Re_{0}^{S} > 1$ the disease is persistent. At last, several numerical simulations are presented to demonstrate the findings of the hypothetical investigation results. These simulations served to vividly illustrate and validate the implications derived from the hypothetical analysis.

## Introduction

It would be fantastic for health authorities to be able to predict future outbreaks when an unknown disease first appears and starts to cause infections and fatalities. An important method for understanding how infectious diseases spread is the representation of infectious disease behaviors by dynamical systems^1,2^. It is presently thought that mathematical models have been crucial instruments in qualitative and quantitatively studying the transmission and management of infectious diseases^3^. Numerous academics used theoretical viewpoints and simulations performed numerically to analyze the transmission of various infectious diseases^4–7^.

The mathematical modeling of disease propagation is limited by various restrictions using the deterministic approach. Despite being easy to understand, they offer few details. As far as we know, environmental fluctuations influence the spread of infectious diseases and make it more complicated to foresee their behavior. In such cases, deterministic systems, while able to make very informative forecasts and previsions, are not appropriate enough^8^. So, there is a pressing need for a developed mathematical model that can take into account the randomness effect, especially in the context of a harmful infectious disease^9^. The uncertainty in a process’s progress is described by its stochastic model. Uncertainty is a result of randomness, which is a result of the evolution of the universe, and ignorance, which is a trait of humans.

In recent years, the stochastic perspective for modeling infectious diseases has received a lot of attention in research papers^10–14^. For example, Din and li presented a detailed analysis of a stochastic delayed model which governs the transmission mechanism of the Hepatitis B virus while considering the white noises and the effect of vaccinations^15^. A stochastic delayed VEIC epidemic model with a general incidence rate was considered in^16^. A stochastic hepatitis B model considering a time-delay in the transmission coefficient and immune response class was established by Din et al. in^17^. Nissar and Sabbar provided a new framework for modeling the dynamics of HIV/AIDS infection under antiretroviral therapy which aims to reduce a person's viral load to an undetectable level by tempered stable Lévy jumps^9^.

Several mathematicians have investigated further infectious epidemic models based on the SIR model, which is the simplest epidemic model and consists of three compartments^18,19^. The SIRD model^20,21^, SEIR model^22–24^, SEIS^25^, and MSEIR^26^ models are modifications of SIR that represent diverse epidemiological situations for diseases^27–29^. This paper proposes a new stochastic SIRD epidemic model that incorporates a mean-reverting Ornstein Uhlenbech process (MROU process). The stochastic version of this model incorporates randomness into the system, allowing for more realistic simulations of disease spread. Stochastic epidemic SIRD models have been used to investigate a variety of diseases including influenza, HIV/AIDS, Ebola, and COVID-19.

The Ornstein-Uhlenbech process (OU process) is the main structural element of the Barndroff-Nielsen-Shephard stochastic volatility model^30,31^. The most recent research has seen huge interest in modeling, which is based on MROU processes of the diffusion type. Larribi et al. investigated a new MROU-based stochastic SIRS epidemic model^32^. Wang et al. studied a SIS model with the OU process and earn the reproduction number of the model to determine the stochastic extinction and persistence^3^. A stochastic SIRC model based on the OU process was analyzed by Zhiming et al.^33^ Zhou and Shi proposed the stationary distribution and extinction of a stochastic SEIS epidemic model motivated by Black Karasinski process^34^.

Our new proposed model is designed to more accurately capture the complexities of infectious diseases by taking into account the changing behaviors of individuals over time. This study provides important insights into the dynamics of infectious diseases and highlights the need for more accurate modeling techniques to better predict and control the spread of outbreaks. It makes a significant contribution to the science of epidemiology and has significant implications for public health policy. The remaining sections of this article are structured as follows. The stochastic model’s problem formulation using the MROU process is presented in “Problem formulation”. This part demonstrates the existence and uniqueness of the system’s global positive solution. The necessary conditions for the disease’s extinction and persistence are created in “Disease extinction and persistence”. The mathematical simulation of the model utilizing simulated data is shown in “Numerical simulation”. “Conclusion” concludes with some discussion and conclusions.

## Problem formulation

In this study, we introduce a MROU process-based stochastic SIRD epidemic model. The proposed model is designed to better capture the complex dynamics of infectious diseases. The SIRD epidemic model employed in this study is divided into four sections: susceptible people (S), infected people (I), recovered people (R), and deceased people (D). At time t, the associated variable for each compartment is denoted by the letters *S*(*t*)*, I*(*t*)*, R*(*t*)*,* and *D*(*t*), respectively. The population size is calculated by adding these classes together.

$$N\left( t \right) = S\left( t \right) + I\left( t \right) + R\left( t \right) + D\left( t \right)$$

The following ordinary differential equation system describes the traditional SIRD epidemic evolution^20^:

1 $$\left\{ \begin{gathered} dS(t) = - \beta (t)S(t)I(t)dt \hfill \\ dI(t) = \beta (t)S(t)I(t)dt - (\alpha + \gamma )I(t)dt \hfill \\ dR(t) = \alpha I(t)dt \hfill \\ dD(t) = \gamma I(t)dt \hfill \\ \end{gathered} \right.$$

with [*S*(*t*_0_)*, I*(*t*_0_)*, R*(*t*_0_)*, D*(*t*_0_)] as starting condition for the start time *t*_0_. The parameter $\beta$ is the infection rate. The parameters $\alpha ,\,\gamma$ are the recovered and death rate respectively. The infection rate of a disease ($\beta$), defined as the number of new infections for each unit of time, is well-known and significant in mathematical epidemiology. The assumption used by the majority of stochastic epidemic models is that the rate of infection in a random environment is a linear function of Gaussian white noise.

$$\beta \to \beta + \sigma dB(t)$$

where the real-valued Brownian motion *B*(*t*) is defined on a probability space $(\Omega ,{\mathbb{F}},{\mathbb{F}}_{t} ,P)$ and σ is the standard deviation of the noise. The following perturbed model of (1) is suggested:

2 $$\left\{ \begin{gathered} dS(t) = - \beta (t)S(t)I(t)dt - \sigma S(t)I(t)dB(t) \hfill \\ dI(t) = \left( {\beta (t)S(t)I(t) - (\alpha + \gamma )I(t)} \right)dt + \sigma S(t)I(t)dB(t) \hfill \\ dR(t) = \alpha I(t)dt \hfill \\ dD(t) = \gamma I(t)dt \hfill \\ \end{gathered} \right.$$

The other alternative model for $\beta$ in a randomly varying environment, is the MROU process which has the following form:

3 $$d\beta (t) = \theta (\overline{\beta }(t) - \beta (t))dt + \xi dB(t)$$

where $\theta ,\,\overline{\beta }$ and ξ are positive constants. The parameter $\theta$ is the speed of revision. The parameter $\overline{\beta }$ determine the long run mean level of the disease transmission rate $\beta (t)$, and ξ is the intensity of the volatility. The results in references^35^ leads us to obtain the explicit solution as follows:

4 $$\beta (t) = \overline{\beta } + (\beta_{0} - \overline{\beta })e^{ - \theta t} + \xi \int\limits_{0}^{t} {\exp ( - \theta (t - s))} dB(s)$$

which $\beta_{0} = \beta (0)$. It is clear that:

5 $${\rm E}(\beta (t)) = \overline{\beta } + (\beta_{0} - \overline{\beta })e^{ - \theta t}$$

and

6 $${\text{var}} (\beta (t)) = \frac{{\xi^{2} }}{2\theta }(1 - e^{ - 2\theta t} )$$

As is well known the term $\xi \int\limits_{0}^{t} {\exp \left( { - \theta (t - s)} \right)dB(s) \sim N(0,\frac{{\xi^{2} }}{2\theta }} (1 - e^{ - 2\theta t} ))$. Then we have,

$$\xi \int\limits_{0}^{t} {\exp ( - \theta (t - s))} dB(s) = \frac{\xi }{{\sqrt {2\theta } }}\sqrt {1 - e^{ - 2\theta } } \frac{dB(t)}{{dt}},\,a.s.$$

Equation (4) can be rewritten as follows:

7 $$\beta (t) = \overline{\beta } + (\beta_{0} - \overline{\beta })e^{ - \theta t} + \sigma (t)\frac{dB(t)}{{dt}},$$

where $\sigma (t) = \frac{\xi }{{\sqrt {2\theta } }}\sqrt {1 - e^{ - 2\theta t} }$. Submitting (7) in to model (1) one can earn the following nonlinear SDE system:

8 $$\left\{ \begin{gathered} dS(t) = - \left( {\overline{\beta } + (\beta_{0} - \overline{\beta })e^{ - \theta t} } \right)S(t)I(t)dt - \sigma (t)S(t)I(t)dB(t) \hfill \\ dI(t) = \left( {\left( {\overline{\beta } + (\beta_{0} - \overline{\beta })e^{ - \theta t} } \right)S(t)I(t) - (\alpha + \gamma )I(t)} \right)dt + \sigma (t)S(t)I(t)dB(t) \hfill \\ dR(t) = \alpha I(t)dt \hfill \\ dD(t) = \gamma I(t)dt \hfill \\ \end{gathered} \right.$$

Given the proper initial condition $S(t_{0} ) = S_{0} > 0,\,I(t_{0} ) = I_{0} > 0,\,R(t_{0} ) = R_{0} \ge 0,$, and $D(t_{0} ) = D_{0} \ge 0$.

To determine whether the solution is universal and beneficial, we must first investigate the dynamic behavior of an epidemic model. The following theorem will be utilized in this part to show that the model is correctly described and therefore biologically meaningful by ensuring that the solution stays in ∆.

### Theorem 1

There exists a singular solution to system (8) for all t > 0 almost surely (a.s.) with any initial value.

### Proof 1

Let us consider X (t) = (S(t), I(t), R(t), D(t)). The coefficients of the system (8) are locally Lipschitz continuous for all starting values, hence there exists a singular solution X(t) ∈ Γ on t ∈ [0, τ_e_], which τ_e_ is the time of an explosion.

Now we prove that τ_e_ = ∞ almost surely (a.s.), and the solution is global. Let κ_0_ > 0 is large enough to allow each member of X(0) are all in interval $[\frac{1}{{\kappa_{0} }},\kappa_{0} ]$. Define,

$$\rho_{\min } (t) = \min \{ S(t),\,I(t),\,R(t),\,D(t)\}$$

$$\rho_{\max } (t) = \max \{ S(t),\,I(t),\,R(t),\,D(t)\}$$

For any κ ≥ κ_0_ and

$$\tau_{\kappa } = \inf \{ t \in [0,\tau_{e} ):\,\rho_{\min } \le \frac{1}{\kappa }\,or\,\rho_{\max } \ge \kappa \}$$

Consider inf ϕ = ∞ where ϕ presents the empty set. Then τ_κ_ is increasing when κ → ∞. Set τ_∞_ = lim_κ→∞_ τ_κ_, then we derive that τ_∞_ ≤ τ_e_ a.s. It is clear that if τ_∞_ = ∞ a.s., it can be concluded that τ_e_ = ∞ a.s. then X (t) ∈  Γ for all t > 0. Let τ_∞_ ≠ ∞, then, there exists two constants $\hat{\delta }$  > 0 and $\tilde{\upsilon }$ ∈ (0, 1) such that P(τ_∞_ ≤ $\hat{\delta }$) ≥  $\tilde{\upsilon }$. Then,

9 $$\exists \,t_{1} \in {\rm Z},\,t_{1} > t_{0} \,\,s.t.\,\,\,P(\tau_{e} \le \overset{\lower0.5em\hbox{$\smash{\scriptscriptstyle\frown}$}}{\delta } ) \ge \tilde{\upsilon }\,\,\,\,\forall t \ge t_{1}$$

Consider the function $\psi$: R_+_^4^ → R_+_, which is twice differentiable, with the definition below:

$$\psi (X(t)) = (S - 1 - \log S) + (I - 1 - \log I) + (R - 1 - \log R) + (D - 1 - \log D)$$

$\psi$ is nonnegative function. Using the system (8) and Ito formula,

$$d\psi (X(t)) = {\mathcal{L}}\psi (X(t))dt + \sigma (t)I(t)dB(t) - \sigma (t)S(t)dB(t)$$

where

$$\begin{gathered} {\mathcal{L}}\psi (X(t)) = \left( {1 - \frac{1}{S(t)}} \right)\left( { - (\overline{\beta } + (\beta_{0} - \overline{\beta })e^{ - \theta t} )S(t)I(t)} \right) \hfill \\ \,\,\,\,\,\,\,\,\,\,\,\,\,\,\,\,\,\,\,\,\,\,\,\,\, \quad + \left( {1 - \frac{1}{I(t)}} \right)\left( {(\overline{\beta } + (\beta_{0} - \overline{\beta })e^{ - \theta t} )S(t)I(t)} \right) \hfill \\ \,\,\,\,\,\,\,\,\,\,\,\,\,\,\,\,\,\,\,\,\,\,\,\,\, \quad - \left( {1 - \frac{1}{I(t)}} \right)(\alpha + \gamma )I(t) + \left( {1 - \frac{1}{R(t)}} \right)\alpha I(t) \hfill \\ \,\,\,\,\,\,\,\,\,\,\,\,\,\,\,\,\,\,\,\,\,\,\,\,\,\, \quad + \left( {1 - \frac{1}{D(t)}} \right)\gamma I(t) + \sigma^{2} (t)I^{2} (t) + \sigma^{2} (t)S^{2} (t): = \Im \hfill \\ \end{gathered}$$

which is bounded and ℑ ∈ R_+_, then

$$\begin{gathered} \int\limits_{0}^{{\tau_{e} \wedge \overset{\lower0.5em\hbox{$\smash{\scriptscriptstyle\frown}$}}{\delta } }} {d\psi (X(t)) \le } \int\limits_{0}^{{\tau_{e} \wedge \overset{\lower0.5em\hbox{$\smash{\scriptscriptstyle\frown}$}}{\delta } }} \Im dt \hfill \\ \,\,\,\,\,\,\,\,\,\,\,\,\,\,\,\,\,\,\,\,\,\,\,\,\,\,\,\,\,\,\,\quad + \int\limits_{0}^{{\tau_{e} \wedge \overset{\lower0.5em\hbox{$\smash{\scriptscriptstyle\frown}$}}{\delta } }} {\sigma (t)I(t)dB(t)} \hfill \\ \,\,\,\,\,\,\,\,\,\,\,\,\,\,\,\,\,\,\,\,\,\,\,\,\,\,\,\,\,\,\,\quad + \int\limits_{0}^{{\tau_{e} \wedge \overset{\lower0.5em\hbox{$\smash{\scriptscriptstyle\frown}$}}{\delta } }} {\sigma (t)S(t)dB(t)} \hfill \\ \end{gathered}$$

and

10 $$\begin{gathered} {\rm E}\left( {\psi (X(t)} \right) \le {\rm E}\left( {\psi (X(0)} \right) + \Im {\rm E}(.) \hfill \\ \,\,\,\,\,\,\,\,\,\,\,\,\,\,\,\,\,\,\,\,\,\,\,\,\,\,\, \le {\rm E}\left( {\psi (X(0)} \right) + \Im \overset{\lower0.5em\hbox{$\smash{\scriptscriptstyle\frown}$}}{\delta } \hfill \\ \end{gathered}$$

The mathematical expectation is represented by E. Let Ω_υ_: = τ_e_ ≤ $\hat{\delta }$ for υ ≥ υ_1_. From Eq. (9), we have $P(\Omega_{\upsilon } ) \ge \tilde{\upsilon }$. Define

$$\Gamma_{{\tau_{e} }} : = \psi (X(\tau_{e} ))$$

Then

$$\Gamma_{{\tau_{e} }} \ge (\kappa - 1 - \log \kappa ) \wedge (\frac{1}{\kappa } - 1 - \log \kappa ).$$

Hence Eqs. (9) and (10) results:

$$\begin{gathered} {\rm E}(\Gamma_{0} ) + \Im \overset{\lower0.5em\hbox{$\smash{\scriptscriptstyle\frown}$}}{\delta } \ge {\rm E}(I_{{\Omega_{\upsilon } }} \Gamma_{{t_{\kappa } }} ) \hfill \\ \,\,\,\,\,\,\,\,\,\,\,\,\,\,\,\,\,\,\,\,\,\,\,\,\,\,\, \ge \tilde{\upsilon }[(\kappa - 1 - \log \kappa ) \wedge (\frac{1}{\kappa } - 1 - \log \kappa )] \hfill \\ \end{gathered}$$

which I_Ωτ_ is the indicator of set Ω_τ_. If υ → ∞ then ∞ > E(Γ_0_) + ℑ$\hat{\delta }$ = ∞ which is contradiction. Then the hypothesis P(τ_∞_ ≤ $\hat{\delta }$ ) > $\tilde{\kappa }$ is incorrect and τ_∞_ = ∞ a.s.

## Disease extinction and persistence

This section examines the factors that will determine whether the disease will disappear or continue to exist. According to the references^32^, the basic reproduction number of the relevant deterministic model of (8) is provided by:

$$\Re_{0} = \frac{{\overline{\beta }N}}{\alpha + \gamma }$$

This controls when an epidemic spreads or the disease just quietly disappears. Using this the threshold of stochastic model (8) is defined as follows^36^:

$$\Re _{{_{0} }}^{S} = \Re _{0} - \frac{{\xi ^{2} N^{2} }}{{4\theta (\alpha + \gamma )}}$$

### Theorem 2

Let X (t) = (S(t), I(t), R(t), D(t)) represent the solution to the system (8) with initial values X(0) ∈ ∆. If $\Re_{{_{0} }}^{S} < 1$ or $\frac{{\overline{\beta }^{2} \theta }}{\alpha + \gamma } < \xi^{2}$ then $P(\mathop {\lim \sup }\limits_{t \to \infty } \frac{\log I(t)}{t} < 0) = 1$.

Specifically, the disease will almost surely become extinct exponentially.

### Proof 2

We have using Ito formula,

11 $$\begin{gathered} d(\log I(t)) = \left( {\left( {\overline{\beta } + (\beta_{0} - \overline{\beta })e^{ - \theta t} } \right)S(t) - (\alpha + \gamma ) - \frac{1}{2}\sigma^{2} (t)S^{2} (t)} \right)dt \hfill \\ \,\,\,\,\,\,\,\,\,\,\,\,\,\,\,\,\,\,\,\,\,\,\,\, \quad + \, \sigma (t)S(t)dB(t) \hfill \\ \,\,\,\,\,\,\,\,\,\,\,\,\,\,\,\,\,\,\,\,\,\,\,\,\, = \left( {f(S) + g(S,t)} \right)dt + \sigma (t)S(t)dB(t) \hfill \\ \end{gathered}$$

where

12 $$f(S) = \overline{\beta }S - (\alpha + \gamma ) - \frac{{\xi^{2} }}{4\theta }S^{2}$$

and

13 $$g(S,t) = (\beta_{0} - \overline{\beta })e^{ - \theta t} S + \frac{{\xi^{2} }}{4\theta }e^{ - 2\theta t} S^{2}$$

We get the following results by integrating from 0 to t and dividing by t:

14 $$\frac{\log I(t)}{t} = \frac{\log I(0)}{t} + \frac{{\int\nolimits_{0}^{t} {f(u)du} }}{t} + \frac{{\int\nolimits_{0}^{t} {g(S,u)du} }}{t} + \frac{\Phi (t)}{t}$$

where $$\Phi (t) = \int\nolimits_{0}^{t} {\sigma (u)S(u)dB(u)}$$.

Since f is increasing function on $(0,\frac{{2\overline{\beta }\theta }}{{\xi^{2} }})$, we earn:

$$\begin{gathered} f(S) \le f(N) = \left( {\overline{\beta }N - (\alpha + \gamma ) - \frac{{\xi^{2} }}{4\theta }N^{2} } \right) \hfill \\ \,\,\,\,\,\,\,\,\,\,\,\,\,\,\,\,\,\,\,\,\,\,\,\,\,\,\,\,\,\,\, = (\alpha + \gamma )\left( {\frac{{\overline{\beta }N}}{\alpha + \gamma } - \frac{{\xi^{2} }}{4\theta (\alpha + \gamma )}N^{2} - 1} \right) \hfill \\ \,\,\,\,\,\,\,\,\,\,\,\,\,\,\,\,\,\,\,\,\,\,\,\,\,\,\,\,\,\,\,\, = (\alpha + \gamma )(\Re_{0}^{S} - 1) \hfill \\ \end{gathered}$$

If $\Re_{0}^{S}$ < 1 then it implies that $\mathop {\lim }\limits_{t \to \infty } \frac{{\int\limits_{0}^{t} {f(u)du} }}{t} = 0$.

However, we also have:

$$\begin{gathered} \int\limits_{0}^{t} {g(S(u),u)du} = \int\limits_{0}^{t} {\left( {(\beta_{0} - \overline{\beta })e^{ - \theta u} S(u) + \frac{{\xi^{2} }}{4\theta }e^{ - 2\theta u} S^{2} (u)} \right)} du \hfill \\ \,\,\,\,\,\,\,\,\,\,\,\,\,\,\,\,\,\,\,\,\,\,\,\,\,\,\,\,\,\,\,\,\,\, \le (\beta_{0} - \overline{\beta })N\int\limits_{0}^{t} {e^{ - \theta u} du + \frac{{\xi^{2} }}{4\theta }N^{2} } \int\limits_{0}^{t} {e^{ - \theta u} du} \hfill \\ \,\,\,\,\,\,\,\,\,\,\,\,\,\,\,\,\,\,\,\,\,\,\,\,\,\,\,\,\,\,\,\,\,\,\, = (\beta_{0} - \overline{\beta })N(1 - \frac{1}{\theta }e^{ - \theta t} ) + \frac{{\xi^{2} }}{4\theta }N^{2} \left( {\frac{ - 1}{{2\theta }}(1 - e^{ - 2\theta t} )} \right) \hfill \\ \end{gathered}$$

Which implies that $\mathop {\lim }\limits_{t \to \infty } \frac{{\int\limits_{0}^{t} {g(S(u),u)du} }}{t} = 0$.

Finally, we have established that $\Phi (t)$ is a local martingale and,

$$(\Phi (t),\Phi (t)) \le \frac{{\xi^{2} }}{2\theta }N^{2} t.$$

From references^36^ it can be established that $\lim_{t \to \infty } \frac{\Phi (t)}{t} = 0$ then, $\lim \sup_{t \to \infty } \frac{\log I(t)}{t} = 0$. Also, we have, $f(S) = - \frac{{\xi^{2} }}{4\theta }(S - \frac{{2\overline{\beta }\theta }}{{\xi^{2} }})^{2} + \frac{{\overline{\beta }\theta }}{{\xi^{2} }} - (\alpha + \gamma ) \le \frac{{\overline{\beta }^{2} \theta }}{{\xi^{2} }} - (\alpha + \gamma )$.

Under condition of theorem, it is clear that,

$$\mathop {\lim \sup }\limits_{t \to \infty } \frac{\log I(t)}{t} \le \frac{{\overline{\beta }^{2} \theta }}{{\xi^{2} }} - (\alpha + \gamma ) < 0.$$

From an epidemiological standpoint, it is more necessary to research the condition that contributes to the disease’s persistence in a community. Following this section, looks into the possibility that the disease will persist.

### Theorem 3

For any initial value X(0) ∈ ∆ if $\Re_{{_{0} }}^{S} > 1$, then $\mathop {\lim \inf }\limits_{t \to \infty } I(t) = \infty$. It means that the disease will persist.

### Proof 3

It is simple to demonstrate from Eq. (12) that:

$$f(S) \ge f(N) - \left( {\overline{\beta } - \frac{{\xi^{2} }}{4\theta }(S + N)} \right)(N - S)$$

From formula (11) we earn:

$$d(\log I(t)) \ge \left( {f(N) - \left( {\overline{\beta } - \frac{{\xi^{2} }}{4\theta }(S + N)(N - S) + g(t,S)} \right)} \right)dt + \sigma (t)S(t)I(t)dB(t)$$

Integrating the two sides from 0 to t gives us,

15 $$\begin{gathered} \log I(t) \ge \log I(0) + f(N)t - (\overline{\beta } - \frac{{\xi^{2} }}{4\theta })Nt + (\overline{\beta } - \frac{{\xi^{2} }}{4\theta }N)\int\limits_{0}^{t} {S(u)du} \hfill \\ \,\,\,\,\,\,\,\,\,\,\,\,\,\,\,\,\,\, + \int\limits_{0}^{t} {g(S,u)du} + \int\limits_{0}^{t} {\sigma (u)S(u)I(u)dB(u)} \hfill \\ \end{gathered}$$

Since $(\overline{\beta } + (\beta_{0} - \overline{\beta }))e^{ - \theta t} \le \overline{\beta } \vee \beta_{0}$***,*** and from the first equation in system (8), the following inequality can be concluded:

$$dS \ge - (\overline{\beta } \vee \beta_{0} )NSdt - \sigma (t)S(t)I(t)dB(t)$$.

Integrating between 0 and t, we earn:

16 $$\int\limits_{0}^{t} {S(u)du} \ge ((\overline{\beta } \vee \beta_{0} )N)^{ - 1} \left( {S(0) - S(t) - \int\limits_{0}^{t} {\sigma (u)S(u)I(u)dB(u)} } \right)$$

Combining Eqs. (15) and (16) implies that,

17 $$\log I(t) \ge f(N)t - (\overline{\beta } - \frac{{\xi^{2} }}{4\theta })Nt + \Theta (t)$$

where

$$\begin{gathered} \Theta (t) = \log I(0) + (\overline{\beta } - \frac{{\xi^{2} }}{4\theta }N)\left( {((\overline{\beta } \vee \beta_{0} )N)^{ - 1} \left( {S(0) - S(t) - \int\limits_{0}^{t} {\sigma (u)S(u)I(u)dB(u)} } \right)} \right) \hfill \\ \,\,\,\,\,\,\,\,\,\, + \int\limits_{0}^{t} {g(S,u)du} + \int\limits_{0}^{t} {\sigma (u)S(u)I(u)dB(u)} \hfill \\ \end{gathered}$$

For local martingales, the law of large numbers yields that $\mathop {\lim }\limits_{t \to \infty } \frac{\Theta (t)}{t} = 0,$ a.s. then

18 $$\mathop {\lim \inf }\limits_{t \to \infty } \frac{\log I(t)}{t} \ge (1 - \alpha - \gamma )f(N).$$

Moreover $f(N) = (\alpha + \gamma )(\Re_{0}^{S} - 1)$**,** then if $\Re_{0}^{S} > 1$ we have:

19 $$\mathop {\lim \inf }\limits_{t \to \infty } \frac{\log I(t)}{t} \ge (1 - \alpha - \gamma )(\alpha + \gamma )(\Re_{0}^{S} - 1) > 0,\,a.s.$$

Hence, $$\mathop {\lim }\limits_{t \to \infty } I(t) = \infty$$.

## Numerical simulation

A simulation study is carried out in this phase to evaluate the proposed model’s proficiency. An approximate diffusion system (8) can be used to demonstrate the analytical findings reported in the preceding sections. Here, we use the Euler approach for the simulation study. Three data sets corresponding to the model (8) with considering the different values of parameters are considered in Examples 1–3. In all examples the values of $S(0) = 0.75,\,I(0) = 0.25,\,R(0) = 0,\,D(0) = 0,\,\beta_{0} = 0.15$*,* are chosen to be same.

### Example 1

**Figure 1:**
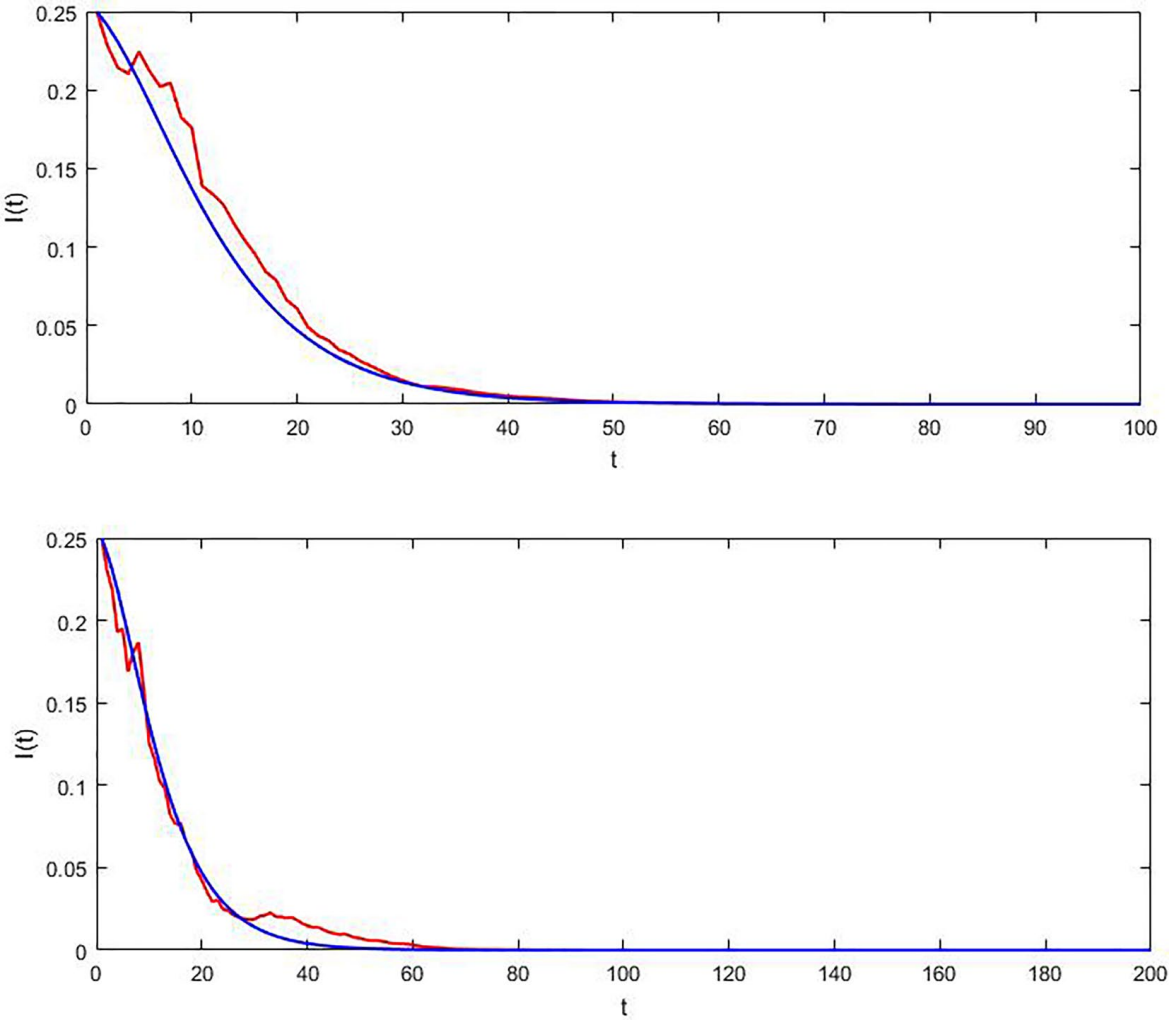
The trajectory of I(t) for the SDE model (red) and the associated deterministic variant (Blue) in the case of $\Re_{0}^{S} < 1$ over various time intervals.

Generate the data sets from the model (8) with various parameter values as follows, $\overline{\beta } = 0.22,\,\theta = 0.4,\,\xi = 0.25,\,\alpha = 0.1,\,\gamma = 0.1,\,\Re_{{^{0} }}^{S} = 0.904 < 1$ is obtained by straightforward calculation, and the condition of theorem 2 is tested. According to theorem 2 for the stochastic model, the disease will be removed from the population. Figure 1 shows the numerical simulation of this example and confirms the results of theorem 2. As we see, the disease will expire in about 50 days.

### Example 2

**Figure 2:**
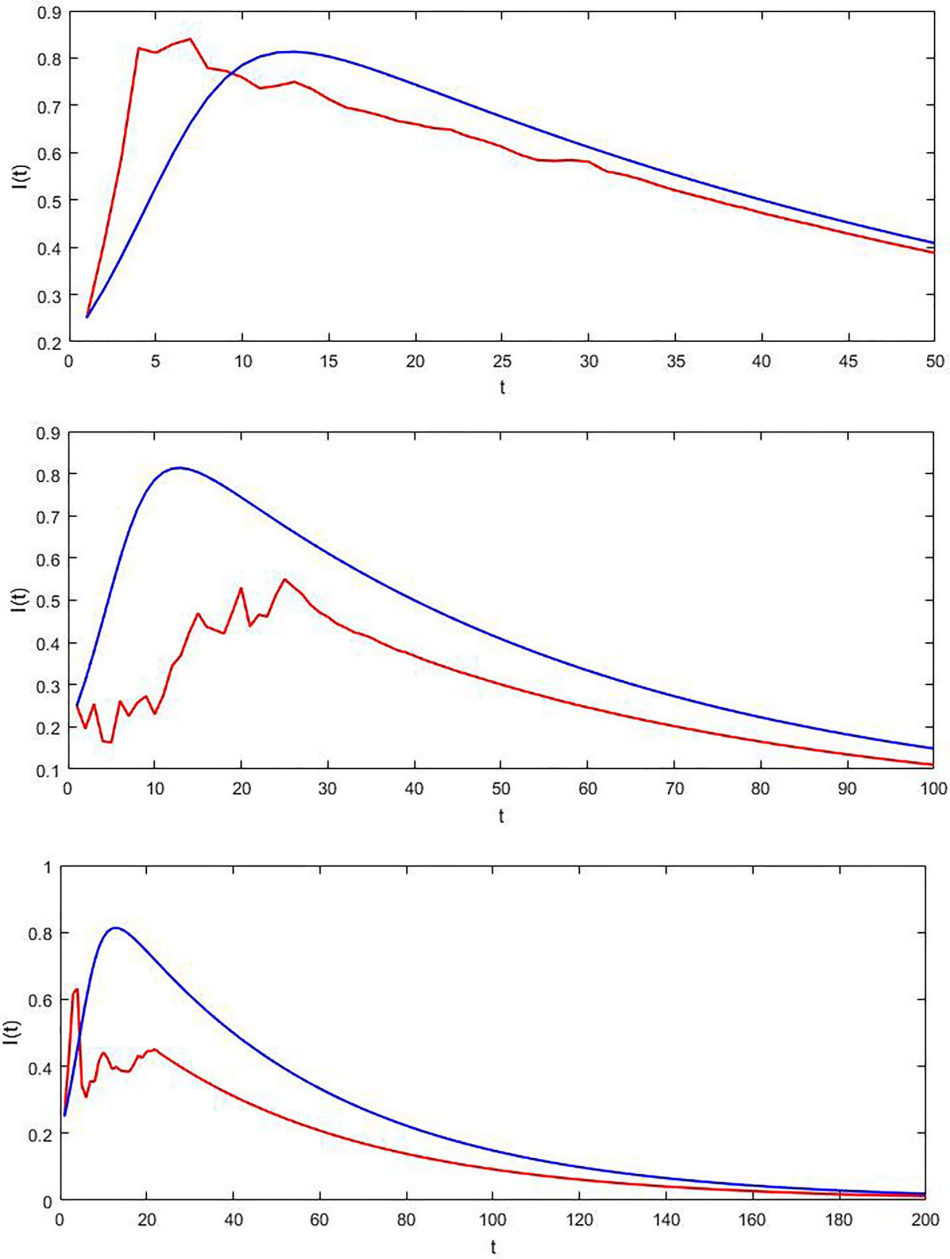
The trajectory of I(t) for the SDE model (red) and the associated deterministic variant (Blue) in the case of $\Re_{0}^{S} > 1$ over various time intervals.

Using the parameter values $\overline{\beta } = 0.35,\,\theta = 1.3,\,\xi = 1.2,\,\alpha = 0.01,\,\gamma = 0.01$. It is easy to calculate that $\Re_{{^{0} }}^{S} = 3.65 > 1$, then the condition of theorem 3 is verified. It means that the disease will persist. In Fig. 2 these results were be shown.

### Example 3

**Figure 3:**
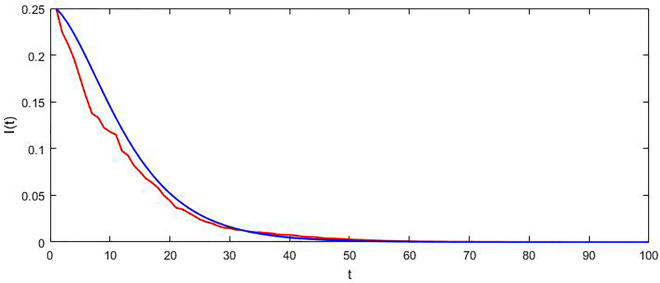
The trajectory of I(t) for the SDE model (red) and the associated deterministic variant (Blue) in the case of $\Re_{0}^{S} = 1$.

This example considers the parameter values $\overline{\beta } = 0.22,\,\theta = 0.13,\,\xi = 0.12,\,\alpha = 0.1,\,\gamma = 0.09\,$ that caused $\Re_{{^{0} }}^{S} = 1$. Numerical simulations in Fig. 3 shows that the disease will be extinct in this critical situation. The scope of the authors’ next research activity is proof of this result*.*

## Conclusion

This study proposes and evaluates a new stochastic SIRD epidemic model using an MROU process and a general non- linear incidence rate. In the suggested model, the existence and uniqueness of a positive global solution were verified. The stochastic system (8) has a threshold $\Re_{0}^{S}$ that controls the extinction and persistence of epidemic diseases. In Theorem 2, we established that under a few additional conditions, when $\Re_{0}^{S} < 1$ the disease disappears exponentially with probability one. Theorem 3 allowed us to demonstrate that the stochastic process I(t) is persistent in mean if $\Re_{0}^{S} > 1$*.* The efficiency and correctness of the current work are demonstrated using numerical simulations with simulated data. The findings of Example 3 show that the disease will expire when the threshold $\Re_{0}^{S} = 1$. Proof of this result is desirable for future research. The other strategy of this research is to investigate the extended Kalman filter for the stochastic SIRD model with the OU process in the future.

## Data Availability

The datasets generated and/or analyzed during the current study are available from the corresponding author upon reasonable request.

## References

[1] Babaei, A., Jafari, H., Banihashemi, S. & Ahmadi, M. Mathematical analysis of a stochastic model for spread of Coronavirus. *Chaos Solitons Fract. 145* (2021). 10.1016/j.chaos.2021.110788 PMC7894125 33642704

[2] Babaei, A., Ahmadi, M. & Jafari, H. A mathematical model to examine the effect of quarantine on the spread of coronavirus. *Chaos Solitons Fract.* **142** (2021). 10.1016/j.chaos.2020.110418 PMC7703523 33288973

[3] Wanga W, Cai Y, Ding Z, Gu Zh (2018). A stochastic differential equation SIS epidemic model incorporating Ornstein Uh lenbeck process. Phys. A.

[4] Emvudu Y, Bongor D, Koïna R (2016). Mathematical analysis of HIV/AIDS stochastic dynamic models. Appl. Math. Model..

[5] Jajarmi, A., Ghanbari, B. & Baleanu, D. A new and efficient numerical method for the fractional modeling and optimal control of diabetes and tuberculosis co-existence. *Chaos Interdiscip. J. Nonlinear Sci.* (2019). 10.1063/1.5112177 31575146

[6] Khan, M. A., Hammouch, Z. & Baleanu, D. Modeling the dynamics of hepatitis E via the Caputo Fabrizio derivative. *Math. Modell. Nat. Phen.* **14** (2019).

[7] Sajjadi, S., Baleanu, D., Jajarmi, A. & Mohammadi Pirouz, H. A new adaptive synchronization and hyper chaos control of a biological snap oscillator. *Chaos Solitons Fract.* **138** (2020).

[8] Kiouach, D., Azami El-idrissi, S. E. & Sabbar, Y. An improvement of the extinction sufficient conditions for a higher-order stochastically disturbed AIDS/HIV model. *Appl. Math. Comput.* **447** (2023).

[9] Nisar KS, Sabbar Y (2023). Long-run analysis of a perturbed HIV/AIDS model with antiretroviral therapy and heavy-tailed increments performed by tempered stable Lévy jumps. Alex. Eng. J..

[10] Ivorraa, B., Ferrández, M.R., Vela-Pérez a, M. & Ramos, A. M. Mathematical modeling of the spread of the coronavirus disease 2019 (COVID-19) taking into account the undetected infections: The case of China. *Commun. Nonlinear Sci. Numer. Simul.* (2020). 10.1016/j.cnsns.2020.105303 PMC7190554 32355435

[11] Tilahun GT, Demie S, Eyob S (2020). Stochastic model of measles transmission dynamics with double dose vaccination. Infect. Dis. Model..

[12] Khoshnaw SHA, Salih RH, Sulaimany S (2020). Mathematical modeling for coronavirus disease in predicting future behaviors and sensitivity analysis. Math. Model. Nat. Phenom..

[13] Sarkar K, Khajanchi S, Nieto JJ (2020). Modeling and forecasting the COVID-19 pandemic in India. Chaos Solitons Fract.

[14] Maleki M, Mahmoudi MR, Heydari MH, Pho KH (2020). Modeling and Forecasting the Spread and Death Rate of Coronavirus (COVID-19) in the World using Time Series Models. Chaos Solitons Fract..

[15] Din A, Li Y (2022). Mathematical analysis of a new nonlinear stochastic hepatitis B epidemic model with vaccination effect and a case study. Eur. Phys. J. Plus.

[16] Din A (2021). The stochastic bifurcation analysis and stochastic delayed optimal control for epidemic model with general incidence function. Chaos.

[17] Din A, Li Y, Yusuf A (2021). Delayed hepatitis B epidemic model with stochastic analysis. Chaos Solitons Fract..

[18] Ariful KM, Kuga K, Tanimoto J (2019). Analysis of SIR epidemic model with information spreading of awareness. Chaos Solitons Fract..

[19] Parsamanesh M, Farnoosh R (2018). On the global stability of the endemic state in an epidemic model with vaccination. Math. Sci..

[20] Fanelli D, Piazza F (2020). Analysis and forecast of COVID-19 spreading in China, Italy and France. Chaos Solitons Fract..

[21] Sebbagh A, Kechida S (2022). EKFSIRD model algorithm for predicting the coronavirus (COVID19) spreading dynamics.

[22] Jiao J, Liu Z, Cai Sh (2020). Dynamics of an SEIR model with infectivity in incubation period and homestead-isolation on the susceptible. Appl. Math. Lett..

[23] Aron JL, Schwartz IB (1984). Seasonality and period-doubling bifurcations in an epidemic model. J. Theoret..

[24] Lu G, Lu Z (2018). Global asymptotic stability for the SEIRS models with varying total population size. Math. Bio sci..

[25] Li MY, Muldowney JS (1995). Global stability for the SEIR model in epidemiology. Math. Biosci..

[26] Asfour HA, Ibrahim M (2015). On the differential fractional transformation method of MSEIR epidemic model. Int. J. Comput. Appl..

[27] Dantas E, Tosin M, Cunha A (2018). Calibration of a SEIRSEI epidemic model to describe the Zika virus outbreak in Brazil. Appl. Math. Comput..

[28] Sun GQ, Xie JH, Huang SH, Jin Z, Li MT (2017). Transmission dynamics of cholera: Mathematical modeling and control strategies. Commun. Nonlinear Sci. Numer. Simul..

[29] Liu X, Stechlinski P (2015). Application of control strategies to a seasonal model of chikungunya disease. Appl. Math. Model..

[30] Bandroff-Nielsen OE, Shephard N (2001). Non-Gaussian OU based models and some of their uses in financial economics. J. R. Stat. Soc. Ser. B.

[31] Zhang V, Yuan R (2021). Pullback attractor for random chemostat model driven by colored noise. Appl. Math. Lett..

[32] Laaribi A, Boukanjime B, Khalifi M, Bouggar D, El Fatini M (2023). A generalized stochastic SIRS epidemic model incorporating mean-reverting OrnsteinUhlenbeck process. Phys. A J..

[33] Zhiming N, Daqing J, Zhongwei C, Xiaojie M (2023). Analysis of Stochastic SIRC model with cross immunity based on Ornstein Uhlenbeck process. Qual. Theory Dyn. Syst..

[34] Zhou B, Shi N (2024). Stationary distribution and extinction of a stochastic SEIS epidemic model motivated by Black Karasinski process. Appl. Math. Lett..

[35] Nabati P, Hajrajabi A (2022). Three-factor mean reverting Ornstein-Uhlenbeck process with stochastic drift term innovations: Nonlinear autoregressive approach with dependent error. Filomat.

[36] Cai S, Cai Y, Mao X (2019). A stochastic differential equation SIS epidemic model with two independent Brownian motions. J. Math. Anal. Appl..

